# Effects of the thawing rate and heating temperature on immunoglobulin A and lysozyme activity in human milk

**DOI:** 10.1186/s13006-022-00487-4

**Published:** 2022-07-07

**Authors:** Xuejing Li, Penprapa Siviroj, Jetsada Ruangsuriya, Nitthinan Yousaibua, Krongporn Ongprasert

**Affiliations:** 1 Department of Community Medicine, Faculty of Medicine, Chiang Mai University, 110 Intrawarorot Road, Si Phum Subdistrict, Amphoe Mueang, Chiang Mai, 50200 Thailand; 2 Department of Biochemistry, Faculty of Medicine, Chiang Mai University, Chiang Mai, Thailand; 3 Obstetric Ward, Maharaj Nakorn Chiang Mai Hospital, Chiang Mai, Thailand

**Keywords:** Human milk, Immunoglobulin A, Lysozyme activity, Thawing rate, Warming temperature

## Abstract

**Background:**

The percentage of infants receiving frozen human milk (HM) is increasing. The effects of thawing and warming on the secretory immunoglobulin A (SIgA) level and lysozyme activity in frozen HM should be investigated to identify optimal methods for preserving immune factors in frozen HM.

**Methods:**

Milk samples were collected from 40 mothers with healthy full-term infants who had been lactating for one to six months. The baseline samples were analyzed within 24 h after collection, and the other samples were frozen at -18 °C before analyses. We compared two methods: placing the container overnight in a refrigerator at 4 °C before warming (slow thawing) and immediately thawing in warm water after removing the sample from the freezer (rapid thawing). Additionally, we investigated the effects of the warming temperature by comparing room temperature (25 °C) and physiological temperature (37 °C). The SIgA concentrations and lysozyme activities in the milk samples were determined using ELISA kits and fluorometric lysozyme activity assay kits, respectively.

**Results:**

The SIgA concentrations and lysozyme activity in frozen HM were 16.5–52.1% and 16.8–39.3% lower than those in fresh HM, respectively. The SIgA concentrations in frozen HM were stable during slow thawing at 37 °C (*p* = 0.072) compared with those in fresh HM. The SIgA concentrations and lysozyme activity were maintained at significantly higher levels during slow thawing than during rapid thawing at 25 °C (*p* = 0.002 and *p* < 0.001, respectively). Slow thawing preserved higher SIgA concentrations and lysozyme activity than rapid thawing at 37 °C, but the difference was not significant.

**Conclusions:**

The SIgA level in HM frozen at -18 °C for two months was stable after overnight thawing in the refrigerator (4 °C for 12 h) before warming to 37 °C compared with that in fresh milk. The thawing of HM in the refrigerator overnight (and then warming to 25 °C or 37 °C for 30 min) has the potential to preserve the SIgA concentration and lysozyme activity to a greater extent than heating immediately after removal from the freezer. Broader temperature ranges should be analyzed to determine the temperature that minimizes the losses in SIgA concentration and lysozyme activity in HM.

## Background

The unique nutritional composition and non-nutritive bioactive factors in human milk (HM) promote the adequate growth and healthy development of infants. Furthermore, numerous biologically active proteins and immune factors are essential for infants who are particularly prone to a variety of infectious pathogens because their immune system is immature [1–3]. HM contains all immunoglobulin classes, and secretory immunoglobulin A (SIgA) is the predominant immunoglobulin detected at all stages of lactation, including in colostrum (88.11%), transition milk (68.35%) and mature milk (81.65%) [4]. Additionally, SIgA, which is considered the most important biological property of HM, primarily binds with invading microbes and thus prevents them from reaching the mucosal membranes [1–3]. Lysozyme is one of the major enzymes in HM and is expressed at high levels in HM, which exhibits approximately 3000-fold higher lysozyme activity than does bovine milk [5, 6]. Lysozyme activity produces both anti-inflammatory actions and bactericidal effects by degrading the outer wall of gram-positive bacteria and has the ability to kill gram-negative bacteria synergistically with lactoferrin and SIgA [1]. Furthermore, some studies have shown that lysozyme possesses antifungal and antiviral activities [3, 6, 7]. Lysozyme is highly heat stable under an acidic pH but becomes heat labile under a neutral pH [8, 9]. The manner in which frozen milk is warmed can have a variable impact on immune proteins in frozen HM [10–13]. The different effects are likely due to the magnitude of protein denaturation caused by heat through proteolysis, refolding, or recrystallization [14–16]. The feeding temperatures are also associated with infant health, including feeding tolerance, body temperatures and gastric temperatures [17, 18]. Previous surveys have shown that a wide range of feeding temperatures (22 °C to 46 °C) are used in practice [19]. An optimal thawing method and an ideal warming temperature for preserving the SIgA concentrations and lysozyme activity are difficult to define because the previous study protocols used different storage durations, different numbers of samples exposed to the freeze–thaw cycle, varied freezing rates, and different milk donors between sample groups. Additionally, most studies of donor milk in milk banks were conducted at high temperatures (pasteurization) rather than in a real household setting [9, 10, 13, 20–23]. The related findings are summarized in Table 1.

**Table 1:** Summary of the previous studies examining the effects of heating on the SIgA concentrations and lysozyme activity

Ref	Year	Sample (n)	Current LabCorp Method		Intervention	Effects	
			SIgA	Lysozyme		IgA	Lysozyme
Paulaviciene et al. [23]	2020	42	NS	ELISA using the commercial CircuLex Human Lysozyme ELISA Kit (MBL, Japan)	Comparison of the effects of HoP (62.5 °C for 30 min) with fresh HM	NS	Reduced (*p* = 0.007)
Vieco et al. [22]	2018	Multiple donors from a human milk bank	Duplicate results obtained using a Bio-Plex 200 system instrument (Bio–Rad Hercules, CA, USA) and determined with the Bio-Plex Pro Human Isotyping Assay (Bio–Rad Hercules, CA, USA)	NS	Comparison of the effects of HTST treatments at different temperatures (70, 72, or 75 °C) for different times (5, 10, 15, 20, and 25 s) with HoP, (62.5 °C for 30 min)	Greater IgA retention was observed after any of the HTST treatments than after HoP (*p* < 0.001)	NS
Chang et al. [21]	2013	16	SIgA ELISA kit (K8870; Immundiagnostik AG, Bensheim, Germany)	Lysozyme enzyme immunoassay kit (Biomedical Technologies Inc., Stoughton, MA, USA)	Comparison of the effects of warming at 40 °C with warming at 60 °C	Stable	Stable
Handa et al. [24]	2014	40	SIgA ELISA kit (ALPCO Diagnostics, Salem, NH, USA)	NS	Comparison of the effects of heating between samples thawed at 4 °C for 24 h prior to warming and those immediately thawed and warmed at 37 °C after being removed from the freezer (-20 °C)	Stable	NS
Akinbi et al. [20]	2010	18 (fresh) 15 (pooled specimens of pasteurized milk)	Enzyme-linked immunosorbent assay (ALPACO Diagnostics, Salem, NH)	Anti-human lysozyme (Accurate Chemical and Scientific Corp, New York, NY, USA)	Comparison of the effects of pasteurization (pulse heating at 62.5 °C for 30 min) with fresh HM	Reduced (*p* < 0.0001)	Reduced (*p* < 0.001)
Evans et al. [13]	1978	6 (IgA samples) 9 (Lysozyme samples)	Electroimmunoassay	Electroimmunoassay	Assessment of the effects of pasteurization for 30 min at 60, 62.5, 65, 67.5, and 70 °C	Progressive loss of SIgA with increases in the heating temperature	Progressive loss of lysozyme activity with increases in the heating temperature

*HM* human milk, *HoP* Holder pasteurization, *HTST* high temperature for a short time, *IgA* immunoglobulin A*, NS* not studied, *SIgA* secretory immunoglobulin A

Although international health authorities have provided standard guidelines that recommend the optimal temperatures and storage durations for each type of HM (fresh, thawed, and left-over from a feeding) [25–27], the evidence-based standards for recommending the optimal HM thawing method and feeding temperature for infants are limited. Several methods have been recommended for the thawing of frozen HM, including placing the container in the refrigerator overnight, running it under warm water, setting it in a container of warm water, or using a waterless warmer [25, 28]. Additionally, the recommendations for the feeding temperatures of milk differ and include cold, room temperature and warm [25, 28].

When planning to improve the storage guidelines for HM that would allow critically ill preterm infants and infants who are unable to feed at their mother’s breast to benefit from the advantages of their mother’s milk, it is important to consider the effects of freezing and warming on the SIgA concentration and lysozyme activity under actual conditions used in daily life. The proportion of infants receiving frozen HM is increasing [29, 30]. In a previous study of Australian women, Binns et al. [29] showed that the proportion of mothers expressing breastmilk peaked in the first six weeks after birth, and the authors discovered a 31% increase in this proportion from 1993 to 2003. We compared the methods involving placing the container overnight in a refrigerator at 4 °C before warming (slow thawing (ST)) and immediately thawing in warm water after removing the container from the freezer (rapid thawing (RT)). Additionally, we investigated the effects of the warming temperature by comparing room temperature (25 °C) and physiological temperature (37 °C).

## Methods

### Participants

The participants were recruited between 28 June and 10 July 2021 through study posters displayed in the well-baby clinic and the lactation rooms of four hospitals in Chiang Mai City, Thailand. After interested mothers contacted the study staff via telephone, they were asked a set of questions corresponding to the inclusion and exclusion criteria. Lactating mothers who had given birth to a full-term infant aged one to six months were recruited for this study. The exclusion criteria were as follows: (a) any underlying disease in the mother or her offspring, (b) maternal age < 18 years or > 40 years, and (c) an inability of the mother to travel to our lactation room on her own. All eligible participants were then asked to make an appointment to provide milk samples; the infant was not required to attend. The participants were paid for their travel expenses. Before providing information and breast milk samples, all the participants signed informed consent forms, which were approved by the Research Ethics Committee, Faculty of Medicine, Chiang Mai University (No. 078 / 2021).

### Milk collection and acquisition of milk samples

**Fig. 1:**
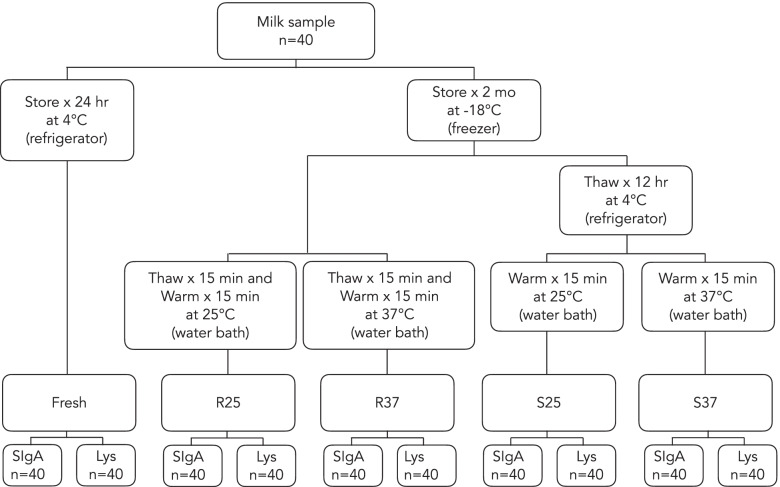
Diagram of the study design. *Lys* lysozyme’ *R25* rapid thawing at 25 °C’ *R37* rapid thawing at 37 °C’ *SIgA* secretory immunoglobulin A’ *ST* slow thawing’ *S25* slow thawing at 25 °C’ *S37* slow thawing at 37 °C

The participants were required to provide milk samples in the lactation room of the Mother and Child Hospital, Chiang Mai City, Thailand. All breast milk samples were obtained between 8:00 a.m. and 11:00 a.m. on the same day using a hospital-grade breast pump (Lactina Electric Selection Pump®, Medela Inc, Switzerland) to ensure sample uniformity. The pump was left on for approximately 15 min or until no additional milk was expressed for at least 5 min. Milk expressed from the left and right breasts was contained in sterile plastic milk bottles and mixed immediately after completion of the breast pumping process (while the milk was still warm and unsettled). The milk was well mixed by rotation before being poured into a 50-mL polypropylene centrifuge tube (Nunc™). Freshly expressed HM specimens were stored in an insulated box with ice packs from the time of collection and aliquoted within 4 h after collection. The milk samples were homogeneously separated into 10-mL aliquots placed in 15-mL polypropylene centrifuge tubes (Nunc™) corresponding to different storage conditions (Fig. 1). Two aliquots were stored at 4 °C from the time of collection and analyzed within 24 h to determine the baseline SIgA concentration and lysozyme activity (fresh). Eight 10-mL aliquots were stored at -18 °C for two months. An estimated effect size and between-group variance for one-way analysis of variance were calculated using the STATA program (Stata Corp. 2019, Stata Statistical Software: Release 16, Stata Corp LLC, College Station, TX, USA). An estimated effect size of 0.25 can be detected with an available sample size of 40 per group investigations, an 80% statistical power level, and a 95% two-tailed confidence level (*α* = 0.05).

### Thawing and warming processes

The thawing and warming processes were conducted according to our preliminary investigation using a digital thermometer (DeltaTrak®, Model 13,309, USA). Rapid thawing was performed by immediately transferring the samples frozen at -18 °C into a temperature-controlled water bath (Memmert Gmbl + Co.KG., West Germany) at either 25 °C or 37 °C and incubating them for 15 min to allow complete thawing. In contrast, slow thawing was performed by placing the frozen samples in a refrigerator (4 °C) for 12 h. The warming process for the rapid thawing of samples was subsequently performed by incubating the samples at either 25 °C or 37 °C for 15 min to equilibrate their temperature. When warming the slow thawing samples, the completely thawed samples were placed into a water bath in which the temperature was controlled at either 25 °C or 37 °C and incubated for 15 min to equilibrate the sample temperature to the set temperature point. Unlike the frozen sample, the milk samples stored in refrigerators at 4 °C were analyzed without thawing or warming. Therefore, our investigated conditions were as follows: rapid thawing at 25 °C (R25), rapid thawing at 37 °C (R37), slow thawing at 25 °C (S25), slow thawing at 37 °C (S37), and unfrozen storage at 4 °C (Fresh) (see Fig. 1).

### Analytical methods

#### SIgA levels

The SIgA levels were determined using ELISA kits (Aviva System Biology, OKEH00516, USA) according to the manufacturer’s protocol. Briefly, the milk samples from each condition were serially diluted up to 200,000 × with deionized water and assay diluent buffer. Both the diluted samples and the SIgA standard were then loaded into each well of the ELISA plate at 100 μL per well. The plate was incubated at 37 °C for 120 min, and the solution was discarded and replaced with biotinylated SIgA detection antibody. The plate was incubated at 37 °C for 60 min, the solution was discarded, and the plate was washed. An avidin-HRP conjugate mixture was added, and the solution was incubated at 37 °C for another 60 min. TMB (tetramethylbenzidine) substrate was added after the solution was discarded and the plate was washed. The plate was then incubated in the dark at 37 °C for 15 min. Subsequently, the stop solution was added, and the absorbance was read at 450 nm using a Synergy H4 Hybrid Reader (Bio-Tek, USA). The SIgA concentrations in the milk samples from each condition were deduced from a SIgA standard curve (0–4000 pg / mL).

### Lysozyme activity

Lysozyme activity in the milk samples was determined with a fluorometric lysozyme activity assay kit (MyBioSource Elabscience®, MBS846601, USA). The milk samples were serially diluted up to 20,000 × with deionizedwater and the assay diluent prior to being loaded into each well of a 96-well plate. The synthetic substrate was then added, and the enzymatic reaction proceeded at 37 °C for 180 min in the dark. The stop solution was added to each reaction well, and the fluorescent product was measured with a Synergy H4 Hybrid Reader (BioTek, USA) using an excitation wavelength of 360 nm and an emission wavelength of 445 nm (Ex / Em = 360 / 445 nm). The amount of fluorescent product was calculated with a standard curve of 4-methylumbelliferone (4-MU) at concentrations ranging from 0 to 100 pmol / well. The activity of lysozyme in each milk sample was subsequently calculated and reported in nmol / min / mg of protein.

### Total protein

The total protein content in the HM samples was determined using Lowry’s method with Folin-Ciocalteu solution (VWR Chemicals, 31,360.264, USA). Each milk sample was diluted 100 × with deionized water, and the diluted sample was mixed with an alkaline solution and the Folin-Ciocalteu solution. The mixture was incubated at room temperature for 10 min, and the absorbance was measured at 650 nm with a Synergy H4 Hybrid Reader (BioTek, USA). The protein concentrations in each milk sample were calculated from a standard curve of bovine serum albumin (BSA) (GE Healthcare, K41–001, USA) at concentrations ranging from 0 to 100 mg / mL.

### Statistical analyses

All statistical analyses were performed using STATA software (Stata Corp. 2019, Stata Statistical Software: Release 16, Stata Corp LLC, College Station, TX, USA). The participant characteristics were described. Continuous variables are presented as the means ± standard deviations (SD), and categorical data are presented as frequencies and percentages. Outliers were detected by constructing boxplots of the SIgA concentrations and lysozyme activity to remove extreme values from the data. The normality of all the parameters was evaluated with the Shapiro–Wilk test. The SIgA concentrations and lysozyme activities were compared between fresh milk and the various frozen HM samples obtained using different thawing methods and warming temperatures, including rapid thawing at 25 °C, rapid thawing at 37 °C, slow thawing at 25 °C, and slow thawing at 37 °C. The SIgA concentrations were compared by one-way analysis of variance (ANOVA) with Tukey’s HSD (honest significance) pairwise comparisons for parametric testing, and lysozyme activities were compared using the Kruskal–Wallis test with Dwass-Steel-Critchlow-Fligner pairwise comparisons for nonparametric testing. The differences in the SIgA concentration or lysozyme activity were considered statistically significant if *p* < 0.05.

## Results

### Demographic characteristics

Forty mothers who had delivered full-term singleton infants provided milk samples. The mean (± SD) values for maternal age and infant age were 28.55 (± 4.77) years and 3.31 (± 0.34) months, respectively. The characteristics of the pregnancies were primiparous (62.5%) and, for the births, vaginal delivery (72.5%). The main characteristics of the mothers and infants are described in Table 2.

**Table 2:** Main characteristics of the participants (*n* = 40)

Characteristics	Mean ± SD or *n* (%)
**Maternal**	
Age (years)	28.55 ± 4.77
BMI (kg / m^2^)	23.83 ± 3.40
Birth order (first / second)	25 (62.5) / 15 (37.5)
Method of delivery (vaginal delivery / Cesarian section)	29 (72.5) / 11 (27.5)
**Infant**	
Age (months)	3.31 ± 0.34
Gestation age (weeks)	38.72 ± 0.96
Birth weight (kg)	3.10 ± 0.34

*BMI* body mass index, *SD* standard deviation

Values are presented as the means ± SDs or numbers (%)

### Effects of different thawing methods and warming temperatures on the SIgA concentrations

**Fig. 2:**
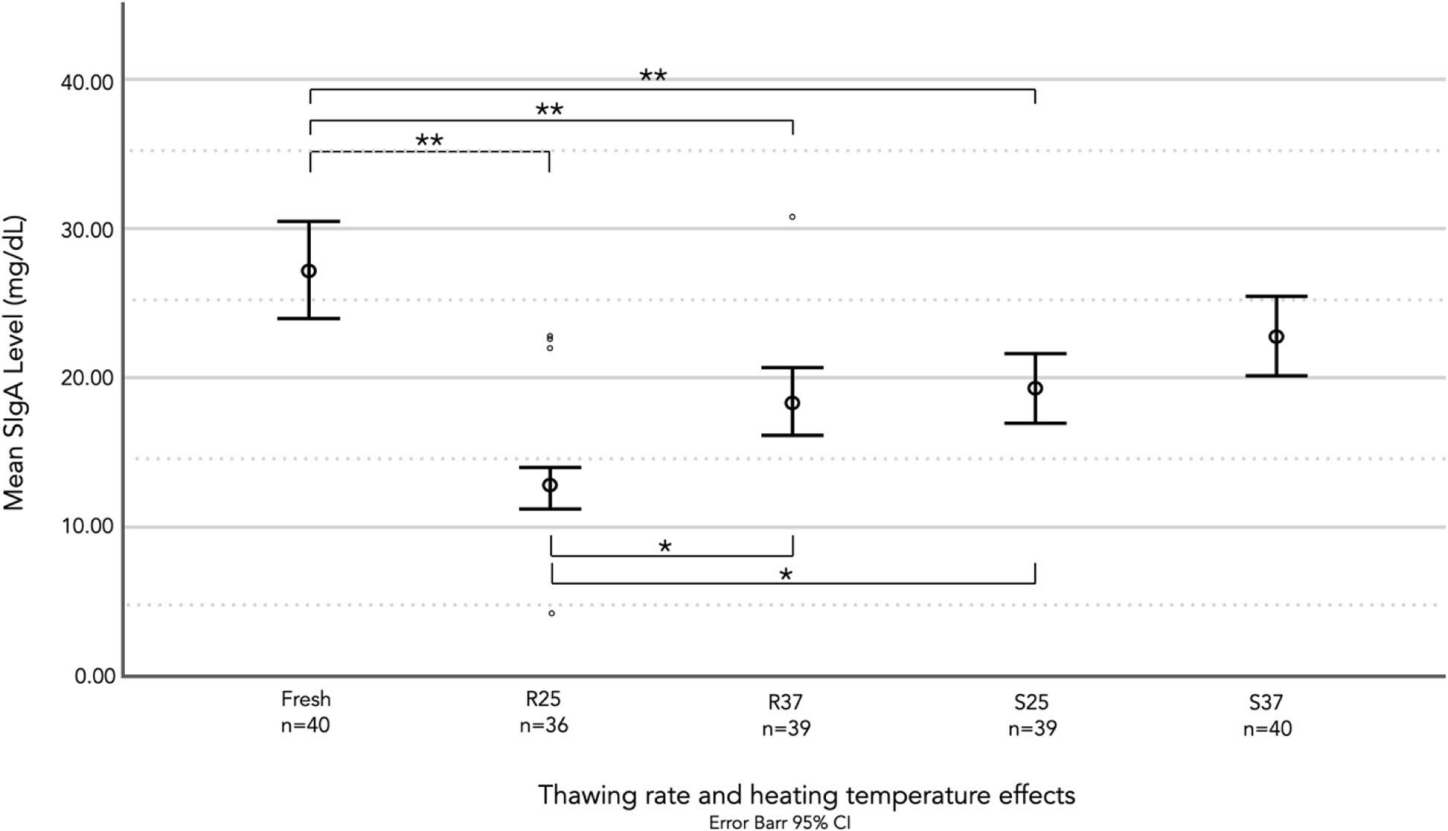
Comparison of the SIgA concentrations between fresh and frozen human milk samples. *R25* rapid thawing at 25 °C, *R37* rapid thawing at 37 °C, *S25* slow thawing at 25 °C, *S37* slow thawing at 37 °C, *SIgA* secretory immunoglobulin A. The data were analyzed by one-way analysis of variance with Tukey’s HSD (honest significance) pairwise comparisons and are presented as the means and 95% confidence intervals (CIs). * *p* < 0.05 and ** *p* < 0.001

A comparison of the SIgA levels between the five different groups is shown in Fig. 2. The SIgA concentration in frozen milk samples decreased significantly during the first two months of freezer storage at -18 °C compared to that of fresh milk (*p* < 0.001), except for the samples that were heated with slow thawing at 37 °C (*p* < 0.072). The mean SIgA concentrations in fresh HM and in HM samples after rapid thawing at 25 °C, rapid thawing at 37 °C, slow thawing at 25 °C, and slow thawing at 37 °C were 27.33, 13.09, 18.72, 19.37, and 22.82 mg / dL, respectively (Table 3). The comparison of the samples obtained after thawing to 25 °C revealed that slow thawing maintained the SIgA concentrations to a significantly greater extent than rapid thawing (*p* = 0.002). An analysis of the rapid methods revealed that warming to physiological temperature (37 °C) maintained the SIgA concentrations more effectively than warming to room temperature (25 °C) (*p* = 0.008) (Table 4).

**Table 3:** SIgA concentrations and lysozyme activity levels detected in fresh and frozen human milk samples

		**Fresh sample**	**Thawing methods and temperature**			
			**Rapid**		**Slow**	
			**25 °C**	**37 °C**	**25 °C**	**37 °C**
**SIgA (mg / dL)**	*N*	40	40	40	39	40
	Mean ± SD (95% CI)	27.33 ± 10.18 (24.07–30.59)	13.09 ± 4.27 (11.73–14.46)	18.72 ± 6.35 (16.69–20.75)	19.37 ± 6.65 (17.21–21.52)	22.82 ± 8.25 (20.18–25.46)
	Minimum–maximum	10.61–46.14	4.51–22.04	7.79–32.98	6.52–37.69	9.40–39.76
	Median	24.99	12.86	18.31	17.76	21.96
	Percentile (25^th^, 75^th^)	19.30, 34.90	10.36, 14.47	14.73, 22.39	15.43, 24.50	16.37, 29.60
	% Decrease	-	52.1	31.5	29.1	16.5
**Lysozyme (nmol / mg protein)**	*N*	39	38	39	40	40
	Mean ± SD (95% CI)	898.64 ± 194.55 (835.58–961.71)	545.74 ± 251.06 (463.22–628.26)	554.54 ± 133.47 (511.27–597.80)	747.77 ± 210.04 (680.60–814.95)	660.55 ± 343.86 (550.58–770.52)
	Minimum–maximum	514.0–1248.0	245.0–1145.0	309.0–873.0	413.0–1486.0	199.0–1653.0
	Median	928	471	535	718.50	659.50
	Percentile (25^th^, 75^th^)	737, 1037	331, 700.75	471, 607	587.25, 842.50	346.25, 926.50
	% Decrease	-	39.3	38.3	16.8	26.6

% Decrease = percent decrease in the mean value compared with the fresh sample

*CI* confidence interval, *SIgA* secretory immunoglobulin A, *SD* standard deviation

**Table 4:** Comparison of SIgA concentrations and lysozyme activities between fresh and frozen human milk samples

**Compared thawing methods**	**SIgA concentration (mg / dL) **^**a**^			**Lysozyme activity (nmol / mg protein) **^**b**^	
	**Mean differences (SD)**	**HSD-test**	**P value**	**W**	***P* value**
Fresh and rapid thawing at 25 °C	14.24 (7.97)	12.113	< 0.001**	-7.69	< 0.001**
Fresh and rapid thawing at 37 °C	8.61 (6.94)	7.325	< 0.001**	-9.05	< 0.001**
Fresh and slow thawing at 25 °C	7.36 (6.01)	6.775	< 0.001**	-4.76	0.007*
Fresh and slow thawing at 37 °C	4.50 (6.07)	3.832	0.072	-4.98	0.004*
Rapid thawing at 25 °C and 37 °C	-5.63 (4.33)	4.787	0.008*	2.14	0.554
Slow thawing at 25 °C and 37 °C	-2.95 (3.47)	2.943	0.395	-2.00	0.619
Rapid thawing at 25 °C and slow thawing at 25 °C	-6.51 (4.94)	5.338	0.002*	5.57	< 0.001**
Rapid thawing at 37 °C and slow thawing at 37°	-4.11 (4.60)	3.493	0.141	0.67	0.990
	Between groups: SS = 4443.320, df = 4, MS = 1110.830; Within groups: SS = 10,665.779, df = 194, MS = 54.978; F = 20.20, *p* < 0.001**			Chi-square = 51.93, df = 4, *p* < 0.001	

^a^ The SIgA concentration data were analyzed by one-way analysis of variance (ANOVA) with Tukey’s HSD pairwise comparisons

^b^ The lysozyme activity data were analyzed using the Kruskal–Wallis test with Dwass-Steel-Critchlow-Fligner pairwise comparisons. Significant differences between fresh milk and frozen HM samples obtained using different thawing methods and warming temperature were identified by * *p* < 0.05 and ** *p* < 0.001

*F* F ratio??? Or is it factor? *df* degrees of freedom, *HSD* (Tukey’s) honest significance test, *HM* human milk, *MS* mean square, *SIgA* secretory immunoglobulin A, *SS* sum of squares, *W* Wilcoxon Z value

A comparison of the attributes of milk samples after thawing and warming processes revealed that slow thawing at 37 °C resulted in the best preservation of the SIgA levels (16.5% decrease), whereas rapid thawing at 25 °C resulted in the greatest reduction in the SIgA levels (52.1%) (Table 3).

### Effects of different thawing methods and warming temperatures on lysozyme activity

**Fig. 3:**
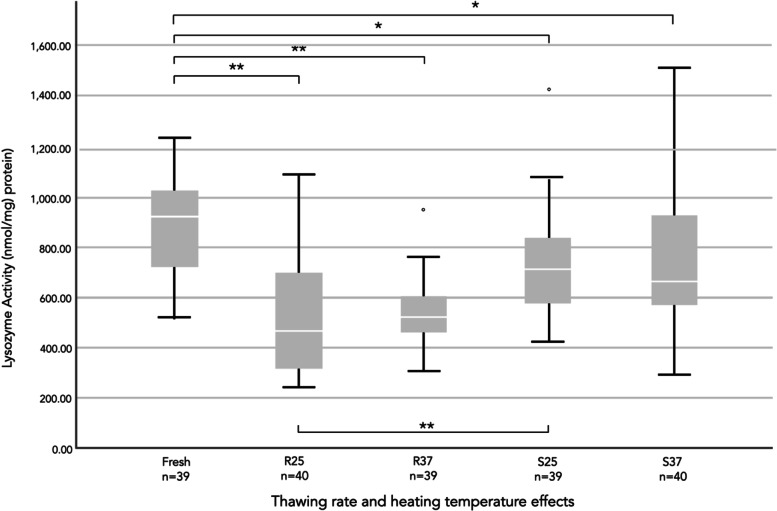
Comparison of lysozyme activities between fresh and frozen human milk samples. *R25* rapid thawing at 25 °C, *R37* rapid thawing at 37 °C, *S25* slow thawing at 25 °C, *S37* slow thawing at 37 °C. The data were analyzed using the Kruskal–Wallis test with Dwass-Steel-Critchlow-Fligner pairwise comparisons. The lysozyme activities are presented as the medians and percentiles (25^th^, 75^th^). * *p* < 0.05 and ** *p* < 0.001

A comparison of lysozyme activity among four different samples of frozen HM revealed significantly decreased activity during the first two months of freezer storage at -18 °C compared with that in fresh milk (Fig. 3). The median lysozyme activity of the HM samples warmed to 25 °C using the rapid thawing method (471.0 nmol / mg protein) was significantly lower than that of samples warmed to 25 °C using the slow thawing method (718.50 nmol / mg protein) (*p* < 0.001) (Table 4). Compared with that of fresh HM, slow thawing at 25 °C resulted in less reduction of lysozyme activity (16.8% decrease), whereas rapid thawing at 25 °C yielded the greatest decrease (39.3% decrease) (Table 3). The warming temperature (25 °C or 37 °C) had no effect on lysozyme activity in samples heated using the same method (rapid or slow thawing).

## Discussion

This study aimed to evaluate the effects of the thawing rate and warming temperature on the stability of SIgA and lysozyme in frozen HM. Our results highlighted that the SIgA level in HM frozen at -18 °C for two months was stable during thawing overnight in a refrigerator (4 °C for 12 h) before warming to 37 °C compared with that in fresh milk. In addition to this method, the SIgA level and lysozyme activity significantly decreased after the heating processes, which was consistent with the results of previous studies showing significant decreases in the SIgA concentration [9, 20, 21] and lysozyme activity [9, 20] following storage and heating processes.

We observed that the thawing of HM overnight in a refrigerator (4 °C for 12 h) before warming preserves the SIgA concentration and lysozyme activity to a greater extent than heating immediately after removal from the freezer through rapid thawing. We found that slow thawing significantly maintained the SIgA levels and lysozyme activity more than rapid thawing at 25 °C. A potential explanation for the finding that slow thawing preserves the SIgA concentrations and lysozyme activity to a greater extent than does rapid thawing is likely due to a slower thawing rate, which minimizes the damage caused by the recrystallization process. Recrystallization exerts additional interfacial tension or shear on the entrapped proteins and causes further damage after the formation of small ice crystals during the freezing process [15]. A comparison of our findings to earlier research is challenging because few studies have examined the impacts of different thawing processes in a household setting. Our findings contradict previous findings that short-term high-temperature pasteurization (72 °C × 5–15 s) preserves SIgA and lysozyme more effectively than longer-term low-temperature pasteurization (62.5 °C × 30 min) [10]. A previous study compared the effect of heating between samples thawed at 4 °C for 24 h prior to warming and samples immediately thawed and warmed at 37 °C after being removed from a freezer (-20 °C) and found no differences in the effects on the SIgA concentrations between the processing methods [24].

The effect of the warming temperature on the SIgA concentration and lysozyme activity was then investigated using rapid and slow thawing methods. The present study shows that rapid warming to physiological temperature (37 °C) significantly preserved a higher SIgA concentration than rapid warming to room temperature (25 °C). A previous study examined changes following warming to 40 °C and 60 °C and found that the higher temperature (60 °C) resulted in a greater decrease in the SIgA concentrations than the lower temperature (40 °C) and that the higher temperature (60 °C) preserved more lysozyme activity than the lower temperature (40 °C), but the differences were not statistically significant [21]. An earlier study also documented progressive decreases in the SIgA concentrations and lysozyme activity during heating at temperatures of 60 °C, 62.5 °C, 65 °C, 67.5 °C, and 70 °C following freezing at -20 °C [13]. Although most of the available evidence indicates that heat causes greater milk protein degradation [31, 32], the opposite result was observed for SIgA levels in our study. This variation might be explained by the temperature used in the study because our study focused on lower temperatures than those used in other studies. Additionally, Akazawa-Ogawa et al. [14] reported that milk proteins exhibit varying degrees of heat stability depending on their structure: for example, each immunoglobulin domain unfolds at a different temperature. As a result, antibodies exhibit a mixture of folded and unfolded structures at different temperatures. These findings may help explain why the effects of temperature on SIgA concentrations are inconsistent and why the reports are highly variable.

### Limitations

Some limitations should be noted. First, the sample size was determined by available resources. Second, our participants were recruited from volunteer samples collected at a single location. Third, each sample contained 10 mL of milk, which is significantly less than the volume of milk that is generally stored and may affect the results. Milk samples with a greater volume should be utilized in future studies to reduce the effects of these variables. Fourth, we investigated two immunological factors (SIgA and lysozyme) even though HM contains other beneficial components, such as cytokines, growth factors, and hormones, for which limited evidence and inconsistent findings are available regarding the effects of heating on bioactive compounds in HM. For example, Escuder-Vieco et al. [22] reported a significantly higher leptin concentration after short-term treatment at high temperature than after Holder pasteurization (HoP), whereas none of the heat treatments exerted a significant effect on the concentrations of adiponectin, ghrelin, epidermal growth factor, or transforming growth factor-beta 2 (TGF-β2). In comparison, an earlier study detected relative increases in the transforming growth factor alpha (TGF-α) and beta 2 (TGF-β2) concentrations in some samples after HoP [33]. Fifth, we studied two different warming temperatures and were unable to determine the best temperature for preserving both the SIgA concentration and lysozyme activity. Therefore, additional research is needed to determine the effects of thawing and warming on a variety of bioactive compounds in HM and to examine a broader range of warming temperatures that are clinically acceptable with the aim of identifying the most suitable warming temperatures for both home-based and hospital settings.

## Conclusions

The SIgA level in HM frozen at -18 °C for two months was stable during thawing overnight in a refrigerator (4 °C for 12 h) before warming to 37 °C compared with that in fresh milk. Thawing HM in a refrigerator overnight and then warming to 25 °C or 37 °C for 30 min has the potential to preserve the SIgA concentrations and lysozyme activity better than heating immediately after removal from the freezer. Further research analyzing a broader range of temperatures is needed to determine the best warming temperature that minimizes decreases in the SIgA concentrations and lysozyme activity in HM.

## Data Availability

Not applicable.

## Abbreviations

BMI: Body mass index
CI: Confidence interval
HM: Human milk
HSD: (Tukey’s) honest significance test
Lys: Lysozyme
HoP: Holder pasteurization
RT: Rapid thawing
R25: Rapid thawing at 25 °C
R37: Rapid thawing at 37 °C
SD: Standard deviation
SIgA: Secretory immunoglobulin A
ST: Slow thawing
S25: Slow thawing at 25 °C
S37: Slow thawing at 37 °C
TMB: Tetramethylbenzidine
